# Case of Puerperal Peritonitis

**Published:** 1865-07

**Authors:** A. Niles

**Affiliations:** Quincy, Ill.


THE
### CHICAGO MEDICAL EXAMINER.

N. S. DAVIS, M.D., Editor.

VOL. VI. JULY, 1865. NO. 7.

### Original Contributions.

### ARTICLE XXV.
### CASE OF PUERPERAL PERITONITIS.

By A. NILES, M.D., Quincy, Ill.

Presented to the Illinois State Medical Society, May, 1865.

*June* 22*d*, 1863, at 6 o'clock P.M., called to attend in labor, Mrs. Dorsey, a primipara, aged twenty-two years, an American by birth.

The labor was terminated by the forceps at 10 o'clock A.M., the next day, the child living, and apparently well. Erysipelas and puerperal peritonitis being rife at the time, the following prescription was ordered:—

℞. Tinct. Ferri Chloridi,------------------ ℥xij

Quinæ Sulphatis,------------------ ℨj

Syr. Simplicis,---------------------- ℥xx

Micæ et Sigma.

Take one teaspoonful three times a day, in one-fourth of a teacup of water.

*June* 25*th*. In the afternoon, a chill occurred, followed by fever and pain in the abdomen. At 6 o'clock P.M., I found the pulse 120; the respiration 40, and entirely thoracic; lochia suppressed, and the lacteal secretion diminished. Prescribed at once, one grain of sulphate of morphia, and directed one-half grain to be taken every hour during the night, and fomentations to be applied to the abdomen.

*June 26th, 8 o'clock A.M.* Found the pain gone; the pulse 100 per minute, and the respiration 12; the lochia returned. Slept some during the night. Continued one-half grain morphia every hour, and take six drops of a mixture containing equal parts syrup of squills and Norwood's tincture of veratrum viride, every three hours, increasing one drop at every repetition. 2 *o'clock P.M.* Symptoms not materially changed, but pulse 80. Continue the treatment. 9 *o'clock P.M.* Respiration 12; pulse 80; vomited several times. Continue the morphia, one-half grain every hour, and diminish the veratrum mixture to six drops every three hours.

*June 27th, 8 o'clock A.M.* Complains of pain in the abdomen, which is tender and tympanitic; pulse 100; respiration 20; the nurse omitted the medicine, because of the vomiting and movements of the bowels. Applied the fomentations, and resumed the morphia, one-half grain every hour, omitting the veratrum. 2 *o'clock P.M.* Pulse 100; pain relieved; respiration 15. 9 *o'clock P.M.* Continue the morphia, and resume the veratrum mixture, six drops every three hours, increasing one drop every successive dose.

*June 28th, 8 o'clock A.M.* The patient vomited several times, and had three operations of the bowels in the night. The nurse again suspended the medicine. Pulse 125 in a minute; respiration 25; abdomen tympanitic and tender. Directed the morphia to be given every hour, one-half grain, and not to be suspended without orders; omit the veratrum. 4 *o'clock P.M.* Dr. Robbins, in consultation, agreed to continue the morphia, and administer the following prescription:—

℞. Ext. Gelsemini semper fluidi,----------

Tinct. Aconiti,------------------------ āā ʒij

Tinct. Veratri Viridis,---------------- ʒj

M. et S.

Take twenty drops every three hours. 9 *o'clock P.M.* Pulse 120; prescribed a powder, composed of pulv. opii gr. vj, quinæ sulphatis gr. ij, repeated every four hours, with twenty-five drops of the veratrum mixture, and two grains of opium in addition, every two hours in the interval, if necessary to relieve the pain.

*June 29th, 8 o'clock A.M.* Nausea and vomiting occurred during the night; tympanites, tenderness, and some pain in the bowels continues; three movements have occurred. Continue the opium, and omit the veratrum mixture. 2 *o'clock P.M.* The patient has but little pain; the nausea has subsided. Directed beef-tea for nourishment. 8 *o'clock P.M.* Pulse 100; respiration 18. Administer four grains of opium, and two grains of quinia every two hours, with ten drops of tincture of digitalis; one grain of opium to be given every hour in the interval, if necessary, to relieve the pain.

*June 30th.* Patient slept some during the night, and is feeling better, but complains of ringing in the ears, and fulness of the head; pulse 80; respiration 16; no pain or tenderness of the abdomen; bowels moved twice last night. Continue the medicine as before, and take the beef-tea freely. 2 *o'clock P.M.* Symptoms not materially changed; is not narcotized; takes more nourishment. Continue the treatment.

*July* 1, 8 *o'clock A.M.* Appears better; pulse 80; respiration 16; slept some during the night; bowels moved three times. Continue the opium, quinine, and digitalis as before, with the beef-tea. 8 *o'clock P.M.* No pain; the abdomen tympanitic; pulse and respiration as before. Prescribed a powder composed of five grains of opium, and two grains of sulphate of quinine, to be repeated every two hours, with ten drops of tincture of digitalis.

*July* 2*d*, 8 *o'clock A.M.* Patient better; rested quite well; pulse and respiration as yesterday; lochia continues; lacteal secretion diminished, but not suppressed; bowels moved twice; abdomen tympanitic, but not tender. 2 *o'clock P.M.* Symptoms not materially changed; bowels move too much; add two grains of tannic acid to each of the powders. 8 *o'clock P.M.* Perspiring freely; complains of being weaker; pulse and respiration not changed. Continue the powders of opium, quinia, and tannic acid every two hours, and ten drops of tincture of digitalis every four hours.

*July* 3*d*. Patient complains of pain and tenderness in the right side; slept some during the night; bowels moved once; perspiration continues; pulse 80; respiration 24. Prescribed a powder, to be repeated once in from two to four hours, composed of opium, quinine, and tannic acid, two grains of each, taking care to repeat often enough to relieve the pain, adding ten drops of tincture of digitalis every four hours, and continuing the beef-tea. 2 *o'clock P.M.* Pain and tenderness in the abdomen continues; pulse 100; respiration 24. Prescribed powders, composed of six grains of opium, with two grains of tannic acid and quinine, repeated every two hours, with ten drops of tincture of digitalis; beef-tea and whiskey continued as before.

*July* 4*th*, 8 *o'clock A.M.* Rested well during the night; respiration 16; pulse 80; some tenderness of the abdomen; no pain. Added to the powders of opium and quinine two grains of persulphate of iron, omitting the tannic acid; treatment, otherwise, as before. 8 *o'clock P.M.* Has no pain; pulse 60; respiration 12. Continue the treatment.

*July* 5*th*, 9 *o'clock A.M* Has rested well; no pain; is inclined to sleep; respiration 12; pulse 60; bowels moved once. Directed one powder to be taken every six hours, or oftener, if the pain returns, with ten drops of tincture of digitalis.

*July* 6*th*, 8 *o'clock A.M.* No pain or tenderness; bowels moved twice; perspiration continues; pulse 70; respiration 20. Continue the powders, with the tincture of digitalis, every six hours, and repeat oftener if the pain returns; continue the beef-tea and whiskey every hour. 8 *o'clock P.M.* Has some pain and tenderness in the abdomen; bowels moved twice; pulse 80; respiration 20. Repeat the powders every two hours until the pain is removed.

*July* 7*th.* Rested well after 2 o'clock; bowels moved twice; no pain or tenderness; lochia and lacteal secretion nearly stopped; pulse 80; respiration 20. Continue the powders every six hours, or oftener if the pain should return, with the beef-tea and whiskey.

No record was kept of the symptoms and treatment after this date, but the patient recovered slowly. The convalescence was retarded by a mammary abscess, and by a cough and expectoration, which disappeared after some weeks, under a supporting plan of treatment.

REMARKS.—My first experience with opium in the treatment of puerperal peritonitis, occurred twenty years since, while practicing in Western New York, during an epidemic of erysipelas and puerperal peritonites, which prevailed extensively in that region, at the time. The antiphlogistic treatment, although vigorously pursued, proved entirely unsuccessful, as every patient subjected to it died, as far as I knew. Believing this treatment worse than useless, I determined to try the effects of opium in conjunction with carbonate of soda, thinking that the opium would relieve the pain and render the patient's condition more comfortable; and that the soda might neutralize the poisonous cause which it was supposed might be lactic acid. This patient recovered; but at the time I regarded the soda as the most useful agent. Subsequent experience has taught me to regard opium as the great remedy, commencing with a moderate dose frequently repeated, carefully watching its effects upon the respiration, and increasing the dose until the pain and tenderness are removed. I believe that large doses are safe, provided they do not reduce the respiration too low. In the case reported, it was not at any time reduced lower than twelve in a minute; if necessary to relieve the pain it probably might be safely reduced below that point.

I would not presume to say that blood-letting, purging, and mercury, may not in some cases be useful in this disease, but I must admit that during a practice of thirty years, I have not seen a well-marked case of puerperal peritonitis recover under the antiphlogistic treatment.

Large doses of opium appear to be better borne when combined with quinia, which probably obviates the congestive effects of the opium, besides being useful by its sustaining power. Veratrum viridi, when it does not nauseate, has a beneficial effect in moderating the circulation, but ordinarily I prefer digitalis, as it is not so liable to nauseate, and probably acts as a tonic upon the heart.

